# Supplementary material for: Immunogenicity decay and case incidence six months post Sinovac-CoronaVac vaccine in autoimmune rheumatic diseases patients
Source: Nat Commun. 2022 Oct 3;13:5801. doi: 10.1038/s41467-022-33042-0 (PMC9527375; doi:10.1038/s41467-022-33042-0)
Supplement: Supplementary file 1 — Supplementary Information [file 41467_2022_33042_MOESM1_ESM.pdf]

# STUDY PROTOCOL

**Institution:** Hospital das Clínicas da Faculdade de Medicina da Universidade de São Paulo (HC-FMUSP)

**Main Coordinator**

Prof. Eloisa Bonfa

| | |
|---|---|
| **TITLE** | **Immunogenicity and safety of an inactivated virus vaccine against SARS-CoV-2 in patients with autoimmune rheumatic diseases** |
| **DISEASES** | Systemic Lupus Erythematosus (SLE), rheumatoid arthritis (RA), ankylosing spondylitis (AS), psoriatic arthritis (APs), dermatomyositis / polymyositis (DM/PM), systemic sclerosis (SS), systemic vasculitis, primary Sjögren's syndrome, primary antiphospholipid syndrome (APS) |
| **BACKGROUND** | The COVID-19 pandemic has progressed rapidly around the world, reaching lethality of up to 20% in different regions (Garcia LF, 2020). More serious cases were observed in elderly patients and with comorbidities, particularly in those with chronic cardiovascular or respiratory diseases, diabetes and hypertension (Garcia LF, 2020; Emmi G, 2020). SARS-Cov-2 infection has raised particular concern in patients with autoimmune rheumatic diseases (DRAI) (Garcia LF, 2020; Emmi G, 2020). Due to chronic inflammatory autoimmune dysregulation and the regular use of immunosuppressive drugs (Fernandez-Ruiz R, 2020), these patients were considered to be at high risk of contracting SARS- |

| | |
|---|---|
| | CoV-2 and potentially having a worse prognosis (Fernandez-Ruiz R, 2020).<br>Vaccine response studies are needed to verify the immunogenicity of the COVID-19 vaccine in immunosuppressed patients with rheumatological diseases. In addition, it is relevant to evaluate the safety of the vaccine in these populations. |
| **OUTCOMES** | Primary Outcome Measure:<br>1. Immunogenicity of CoronaVac in a cohort of ARD patients compared with age- and sex-matched controls without these conditions:<br>- Seroconversion rate of anti-SARS-Cov-2 IgG antibodies<br>- Presence of ≥30% of neutralizing activity of SARS-CoV-2 antibodies<br>2. Safety of CoronaVac in ARD patients compared with controls without these conditions<br>3. Immunogenicity of CoronaVac in a cohort of ARD patients compared with age- and sex-matched controls without these conditions 6 months after the second vaccine dose.<br>4. Immunogenicity of an additional 4th dose of a mRNA (BNT162b2) heterologous SARS-CoV-2 vaccine in poor/non-responders ARD patients previously vaccinated with 3rd of a SARS-CoV-2 inactivated vaccine. |
| **SAMPLE SIZE** | 1. **AUTOIMMUNE RHEUMATIC DISEASES (ARD) PATIENTS**<br>The sample size calculation was based on the previous 15% reduction of seroconversion rate after primo vaccination with the 2009 non-adjuvanted influenza A/H1N1 vaccine in a large cohort of ARD patients. Expecting seroconversion rates of 63% in the ARD patient's cohort and 78% in the control group, considering an alpha error of 5% and power of 80%, in 5 : 1 ratio in order to include more ARD patients, the minimum sample required would |

| | |
|---|---|
| | be 445 ARD patients and 89 healthy subjects, sex-matched and with similar ages. Expecting a higher SC rate of 98% for this vaccine, such sample size had a power greater than 99% to detect a 15% reduction in SC of ARD patients. Due to the peak of pandemics ongoing in Brazil during the vaccination period, we invited more patients and controls, expecting a high incidence of previously infected people and a high rate of infections.<br>A sample was calculated for patients with other rare rheumatological diseases, grouped into five diseases (systemic scleroderma, dermatomyositis/myositis, primary Sjogren's syndrome, systemic vasculitis and primary antiphospholipid antibody syndrome) being followed up in Rheumatology outpatient clinic (HCFMUSP). For the calculation, a seroconversion ratio of p1 = 50% is assumed for each disease and p2 = 74.25% as the average seroconversion of healthy controls.<br>The sample size for each rare disease was 50, thus 250 being the number of patients with other diseases.<br><br>2. **CONTROL GROUP:** 271 healthy controls matched for age and sex will be included according to the need for controls for lupus (n = 74), rheumatoid arthritis (n = 61) and ankylosing spondylitis / psoriatic arthritis (n = 136) will be selected to receive the vaccine at HCFMUSP. |
| **STUDY DESIGN** | The study will be prospective observational in a cohort of patients to be vaccinated and that will be compared independently to the healthy, matched control group by sex and age. |
| **POPULATION** | **Inclusion criteria:**<br>- RA patients according to the classification criteria of the European league against rheumatism (EULAR) / American College of Rheumatology (ACR) (Aletaha D, 2010). |

| | |
|---|---|
| | - Patients with spondyloarthritis, more specifically with the diagnosis of axial spondyloarthritis (ASAS criteria 2009) (Rudwaleit M 2009) and psoriatic arthritis (CASPAR 2012 criteria) (Tillet W 2012) will be included in the present study.<br>- SLE patients according to the SLICC classification criteria (Petri M, 2012)<br>- Patients with systemic sclerosis according to the ACR preliminary criteria (Van den Hoogen F et al., 2013)<br>- Adult and pediatric patients with inflammatory myopathies according to the criteria of Bohan and Peter (Bohan A et al., 1975)<br>- Patients with primary vasculitis (Takayasu's arteritis, granulomatosis with polyangiitis, polyarteritis nodosa) (Arend WP et al., 1990; Leavitt RY et al., 1990; Lightfoot RW et al., 1990)<br>- Patients with primary Sjogren's Syndrome (classification criteria of the European Study Group on Diagnostic Criteria for Sjögren's Syndrome) (Vitali C et al., 1996)<br>- Patients with primary APS (Sydney classification criteria) (Miyakis S et al., 2006)<br>- Patients with HIV-related illness<br><br>**Exclusion criteria:**<br>History of anaphylactic response to vaccine components<br>- Acute acute febrile illness<br>- Guillain-Barré syndrome, decompensated heart failure (class III or IV), demyelinating disease.<br>- History of live virus vaccine up to 4 weeks before, virus vaccine inactivated up to 2 weeks before.<br>- History of having received blood products up to 6 months before the study. |

| | |
|---|---|
| | - Individuals who do not accept to participate in the study and / or whose guardians do not agree to participate in the study.<br>- Hospitalized patients<br>- Patients with severe conditions requiring hospitalization<br>- Pre-vaccination positive COVID-19 serology (IgG or neutralization antibodies). |
| **RELEVANT METHODS** | ARD patients and controls will be vaccinated with the vaccine against COVID-19 by HCFMUSP.<br>The vaccination protocol will consist of two doses of the COVID-19 vaccine for patients and controls, in addition to the collection of a blood sample on the day of the 1st vaccine dose (D0), on the day of the 2nd vaccine dose (28 days after first dose) and 40 days after the second dose of the vaccine. An additional (third) dose will be given 6 months after the second dose (D210) and immunogenicity will be assessed 30 days later (D240). Blood collection will also be performed at D210 prior to vaccination (3rd dose) to assess humoral response decay.<br>The serum obtained during collection will be stored in a -70ºC freezer for testing for immunogenicity.<br>Immunogenicity will be assessed using the seroconversion rates of total IgG antibodies against S1 and S2 antigens and neutralizing antibodies to SARS-CoV-2 on the day of the 2nd dose and 40 days after the 2nd dose, using the 2-dose vaccine schedule is 28 days apart. Additionally, immunogenicity will be assessed prior to the third dose vaccination, 6 months after the second dose, and 30 days after the third dose vaccine dose (6 months after the second dose). |
| **SAFETY** | Healthy patients and controls will be advised of the possible side effects of the vaccine. In addition, patients and controls will be |

| | |
|---|---|
| | instructed to note in a standard diary the changes that occur in the period, such as:<br>Local reactions: pruritus, local pain, erythema, induration at the vaccine site,<br>Systemic reactions: tiredness, fatigue, headache, nausea, diarrhea, fever, chills, myalgia, arthralgia and diarrhea, signs of airway infections (cough, sputum, sore throat, ear pain or discharge, nasal congestion or expectoration, dyspnoea, sneezing), fever (axillary temperature> 37.8º C), itchy skin, rash, vertigo, abdominal pain, drowsiness, malaise, flushing, extremity pain / discomfort, back pain, edema, inappetence.<br>In addition, the need for medical care, hospitalizations, severity of infections, sick days, absenteeism at work and treatment received will be raised, regarding a clinical condition compatible with COVID-19.<br>The standardized diary of adverse events will be evaluated by the researchers 28 days after the first dose of vaccine and 40 days after the second dose. The diary includes guidance for telephone communication of any events that require hospital care. If tertiary care is required, the participant will be transferred to our University hospital (Hospital das Clínicas FMUSP). Serious adverse events will be defined as those that result in hospitalization or death. They will be reported to the National Ethics Comitee (CONEP) within 24 hours.<br>Surveillance will be maintained for new cases of COVID-19 through weekly WhatsApp messages. For patients who present symptoms compatible with COVID-19 infection after vaccination, according to the recommended routine for monitoring immunosuppressed patients with a suspicious condition, a nasal swab will be collected to perform PCR for COVID, with sequencing |

| | |
|---|---|
| | for mutant strains in positive cases. for a period of up to 12 months after the 2nd dose of the vaccine. Positive samples will be sequenced by the protocol established at IMT-FMUSP (1) using the MinION platform (Oxford Nanopore Technologies, UK). The extracted RNA is converted into cDNA using the SuperScript IV First-Strand Synthesis System (Thermo Fisher Scientific, USA) and amplification of the complete genome using the Multiplex PCR protocol - version 3, described by Quick 2020 (2). The amplified samples are purified using 1x Ampure XP beads (Beckman Coulter, United Kingdom) and 70% alcohol solution twice. Amplicons are quantified with Qubit dsDNA High Sensitivity Assay Kit fluorophore and assayed in the Qubit 3.0 instrument (Life Technologies, USA (ThermoFisher) as recommended by the manufacturer. Amplicons are normalized to 240 ng and identified with barcodes. the following EXP-NBD104 (1-12) and EXP-NBD114 Native Barcoding Kits (Oxford Nanopore Technologies, UK) and Ligation Sequencing kit (ONT, SQK-LSK109) are used according to protocol (2). The final library is then loaded into the flowcell R9.4.1 and sequenced using the MinION platform (Oxford Nanopore Technologies - ONT) .The programs MinKNOW (version 19.10.1) and RAMPART are used to monitor the sequencing run in real time (https: //artic.network/rampart)<br>After the end of the race, the fast5 files are subjected to base calls and separation of the barcodes (basecalled and demultiplexed) using the Guppy software, version 2.2.7 (Oxford Nanopore Technologies, UK). To obtain the consensus sequences, the fastq5 files are mapped against the reference genome of SARS-CoV-2 isolate Wuhan-Hu 1 (GenBank accession number MN908947) using the program minimap2 (version 2.28.0) and SamTools. |

| | |
|---|---|
| | Bioinformatics analyzes will follow the protocols described by the research group ARTIC (https://artic.network/ncov-2019). |
| **STATISTICAL ANALYSIS** | The results will be presented as mean ± standard deviation (SD) or median for continuous variables and percentage for categorical variables. Data for continuous variables will be compared using Student's t-test or Mann-Whitney to assess differences in immunogenicity (serology) between patients with rheumatic diseases versus healthy controls. Differences between categorical variables (safety and effectiveness) will be assessed using the chi-square or Fisher's exact tests. Statistical significance will be established with p <0.05. |

**REFERENCES**

Arend WP, Michel BA, Bloch DA, et al. The American College of Rheumatology 1990 criteria for the classification of Takayasu arteritis. *Arthritis Rheum* 1990 ; 33 : 1129 – 34 .

Arnett FC, Edworthy SM, Bloch DA, McShane DJ, Fries JF, Cooper NS, *et al*. The American Rheumatism Association 1987 revised criteria for the classification of rheumatoid arthritis. *Arthritis Rheum* 1988; 31: 315-24.

Avelino-Silva VI, Miyaji KT, Hunt PW, Huang Y, Simoes M, Lima SB, et al. CD4/CD8 Ratio and KT Ratio Predict Yellow Fever Vaccine Immunogenicity in HIV-Infected Patients. PLoS Negl Trop Dis 2016; 10(12):e0005219.

Avelino-Silva VI, Miyaji KT, Mathias A, Costa DA, de Carvalho Dias JZ, Lima SB, et al. CD4/CD8 Ratio Predicts Yellow Fever Vaccine-Induced Antibody Titers in Virologically Suppressed HIV-Infected Patients. *J Acquir Immune Defic Syndr* 2016:71(2):189-95.

Bélec L, Authier F, Gherardi RK. Safety and Immunogenicity of Hepatitis B Vaccine Regimens in Adults With HIV-1. *JAMA* 2011;306(2):156–7.

Bode RK, Klein-Gitelman MS, Miller ML, Lechman TS, Pachman LM. Disease activity score for children with juvenile dermatomyositis: reliability and validity evidence. *Arthritis Rheum* 2003; 49: 7-15

Bohan A, Peter JB. Polymyositis and dermatomyositis (first of two parts). *N Engl J Med* (1975) 292(7):344–7.

Bombardier C, Gladman DD, Urowitz MB, Caron D, Chang CH. Derivation of the SLEDAI. A disease activity index for lupus patients. The Committee on Prognosis Studies in SLE. *Arthritis Rheum* 1992; 35: 630-40.

Borba EF, Saad CG, Pasoto SG, Calich AL, Aikawa NE, Ribeiro AC, et al. Influenza A/H1N1 vaccination of patients with SLE: can antimalarial drugs restore diminished response under immunosuppressive therapy? *Rheumatology (Oxford)* 2012;51:1061-9.

BRASIL. Ministério da Saúde. Agência Nacional de Vigilância Sanitária. Relatório: bases técnicas para decisão do uso emergencial, em caráter experimental de vacinas contra a COVID-19. Brasília, 2021

Brown, LB; Spinelli, MA; Gandhi, M. The interplay between HIV and COVID-19: summary of the data and responses to date, *Current Opinion in HIV and AIDS:* 2021;16(1):63-73.

Candido D et al Evolution and epidemic spread of SARS-CoV-2 in Brazil Science,2020: 369: 1255-1260

Consolaro A, Ruperto N, Bazso A, Pistorio A, Magni-Manzoni S, Filocamo G, et al. Development and validation of a composite disease activity score for juvenile idiopathic arthritis. *Arthritis Rheum* 2009;61:658–66.

Deeks SG, Overbaugh J, Phillips A, Buchbinder S. HIV infection. Nat Rev Dis Primers. 2015 Oct 1;1:15035. doi: 10.1038/nrdp.2015.35. PMID: 27188527.

de Medeiros DM, Silva CA, Bueno C, Ribeiro AC, Viana V dos S, Carvalho JF, et al. Pandemic influenza immunization in primary antiphospholipid syndrome (PAPS): a trigger to thrombosis and autoantibody production? *Lupus* 2014;23:1412-6.

Dougados M, van der Linden S, Juhlin R, Huitfeldt B, Amor B, Calin A, Cats A, Dijkmans B, Olivieri I, Pasero G, et al. The European Spondylarthropathy Study Group preliminary criteria for the classification of spondylarthropathy. *Arthritis Rheum* 1991; 34(10): 1218-27.

Emmi G, Bettiol A, Mattioli I, *et al*. SARS-CoV-2 infection among patients with systemic autoimmune diseases. *Autoimmun Rev* 2020;19(7):102575.

Esparza RH, Swaak T, Aarden L, Smeenk R. Complement-fixing antibodies to dsDNA detected by the immunofluorescence technique on Crithidia luciliae. A critical appraisal. *J Rheumatol* 1985; 12: 1109-17.

Fernandez-Ruiz R, Masson M, Kim MY, *et al*. Leveraging the United States Epicenter to Provide Insights on COVID-19 in Patients With Systemic Lupus Erythematosus. *Arthritis Rheumatol* 2020 ;72(12):1971-80.

França IL, Ribeiro AC, Aikawa NE, Saad CG, Moraes JC, Goldstein-Schainberg C, et al. TNF blockers show distinct patterns of immune response to the pandemic influenza A H1N1 vaccine in inflammatory arthritis patients. *Rheumatology (Oxford)* 2012;51:2091-8.

Furer V, Rondaan C, Heijstek MW, Agmon-Levin N, van Assen S, Bijl M, et al. 2019 update of EULAR recommendations for vaccination in adult patients with autoimmune inflammatory rheumatic diseases. *Ann Rheum Dis* 2020;79(1):39-52.

García, L.F. Immune Response, Inflammation, and the Clinical Spectrum of COVID-19. *Front Immunol* 2020;11:1441.

Garrett S, Jenkinson T, Kennedy LG, Whitelock H, Gaisford P, Calin A. A new approach to defining disease status in ankylosing spondylitis: the Bath Ankylosing Spondylitis Disease Activity Index. *J Rheumatol* 1994;21:2286-91.

Gianfrancesco M, Hyrich KL, Al-Adely S, et al. Characteristics associated with hospitalisation for COVID-19 in people with rheumatic disease: data from the COVID-19 Global Rheumatology Alliance physician-reported registry. *Ann Rheum Dis* 2020;79(7):859-66.

Gladman DD, Ibañez D, Urowitz MB. Systemic lupus erythematosus disease activity index 2000. *J Rheumatol* 2002;29(2):288-9.

Gotzsche PC, Hansen M, Stoltenberg M, et al. Randomized, placebo controlled trial of withdrawal of slow-acting antirheumatic drugs and of observer bias in rheumatoid arthritis. Scand J Rheumatol 1996;25:194-9.

Hochberg MC. Updating the American College of Rheumatology revised criteria for the classification of systemic lupus erythematosus. *Arthritis Rheum* 1997;40(9):1725.

Kasukawa RS, Gordon C. Mixed connective tissue disease and anti-nuclear antibodies: proceedings of the International Symposium on Mixed Connective Tissue Disease and Anti-nuclear Antibodies, Tokyo, 29-30 August 1986/editors, Reiji Kasukawa, Gordon C. Sharp. In International Symposium on Mixed Connective Tissue Disease and Anti-nuclear Antibodies 1986: Tokyo, Japan: Amsterdam; New York: Excerpta Medica; New York, NY, USA: Elsevier Science Pub. Co.; 1987.

Lan, S.H., et al. Tocilizumab for severe COVID-19: a systematic review and meta-analysis. *Int J Antimicrob Agents* 2020;56(3):106103.

Leavitt RY, Fauci AS, Bloch DA, Michel BA, Hunder GG, Arend WP, *et al*. The American College of Rheumatology 1990 criteria for the classification of Wegener's granulomatosis. *Arthritis Rheum* 1990; 33: 1101-7.

Lightfoot RW Jr, Michel BA, Bloch DA, Hunder GG, Zvaifler NJ, McShane DJ, Arend WP, Calabrese LH, Leavitt RY, Lie JT, et al. The American College of Rheumatology 1990 criteria for the classification of polyarteritis nodosa. *Arthritis Rheum* 1990;33(8):1088-93.

Lovell DJ, Lindsley CB, Rennebohm RM et al. Development of validated disease activity and damage indices for the juvenile idiopathic inflammatory myopathies. II. The Childhood Myositis Assessment Scale (CMAS): a quantitative tool for the evaluation of muscle function. The Juvenile Dermatomyositis Disease Activity Collaborative Study Group. *Arthritis Rheum* 1999; 42: 2213-9.

Lukas C, Landewé R, Sieper J, Dougados M, Davis J, Braun J, *et al*. Assessment of Spondylo Arthritis international Society. Development of an ASAS-endorsed disease activity score (ASDAS) in patients with ankylosing spondylitis. *Ann Rheum Dis* 2009; 68: 18-24.

Mikuls, T. R. *et al.* American College of Rheumatology Guidance for the Management of Rheumatic Disease in Adult Patients During the COVID-19 Pandemic: Version 3. *Arthritis Rheumatol.* **73**, e1–e12 (2021).

Miossi R, Fuller R, Moraes JC, Ribeiro AC, Saad CG, Aikawa NE, et al. Immunogenicity of influenza H1N1 vaccination in mixed connective tissue disease: effect of disease and therapy. *Clinics* (Sao Paulo) 2013;68:129-34.

Moll JM, Wright V. Psoriatic arthritis. Semin Arthritis Rheum 1973; 3(1): 55-78.

Mukhtyar C, Lee R, Brown D, Carruthers D, Dasgupta B, Dubey S, *et al*. Modification and validation of the Birmingham Vasculitis Activity Score (version 3). *Ann Rheum Dis* 2009; 68: 1827-32.

Ozen S, Pistorio A, Iusan SM, Bakkaloglu A, Herlin T, Brik R, et al; Paediatric Rheumatology International Trials Organisation (PRINTO). EULAR/PRINTO/PRES criteria for Henoch-Schönlein purpura, childhood polyarteritis nodosa, childhood Wegener granulomatosis and childhood Takayasu arteritis: Ankara 2008. Part II: Final classification criteria. *Ann Rheum Dis* 2010;69:798-806.

Pasoto SG, Ribeiro AC, Viana VS, Leon EP, Bueno C, Neto ML, et al. Short and long-term effects of pandemic unadjuvanted influenza A(H1N1)pdm09 vaccine on clinical manifestations and autoantibody profile in primary Sjögren's syndrome. *Vaccine* 2013; 31:1793-8.

Petty RR, Southwood T, Manners P, Baum J, Glass DN, Goldenberg J, et al. International League of Associations for Rheumatology classification of juvenile idiopathic arthritis: second revision, Edmonton, 2001. *J Rheumatol* 2004; 2:390-2.

Prevoo ML, van't Hof MA, Kuper HH, van Leeuwen MA, van de Putte LB, van Riel PL. Modified disease activity scores that include twenty-eight-joint counts. Development and validation in a prospective longitudinal study of patients with rheumatoid arthritis. *Arthritis Rheum* 1995; 38: 44-8.

Quick, Joshua. nCoV-2019 sequencing protocol. Disponível em dx.doi.org/10.17504/protocols.io.bbmuik6w

Ranzani OT, Bastos LSL, Gelli JGM, Marchesi JF, Baião F, Hamacher S, Bozza FA. Characterisation of the first 250 000 hospital admissions for COVID-19 in Brazil: a retrospective analysis of nationwide data. *Lancet Respir Med* 2021; 15:S2213-2600(20)30560-9.

RECOVERY Collaborative Group, Horby, P., et al. Effect of Hydroxychloroquine in Hospitalized Patients with Covid-19. *N Engl J Med* 2020:NEJMoa2022926. doi: 10.1056/NEJMoa2022926;

Ribeiro AC, Guedes LK, Moraes JC, Saad CG, Aikawa NE, Calich AL, et al. Reduced seroprotection after pandemic H1N1 influenza adjuvant-free vaccination in patients with rheumatoid arthritis: implications for clinical practice. *Ann Rheum Dis* 2011;70:2144-7.

Ribeiro AC, Laurindo IM, Guedes LK, Saad CG, Moraes JC, Silva CA, et al. Abatacept and reduced immune response to pandemic 2009 influenza A/H1N1 vaccination in patients with rheumatoid arthritis. *Arthritis Care Res (Hoboken)* 2013;65:476-80.

Rider LG, Koziol D, Giannini EH et al. Validation of manual muscle testing and a subset of eight muscles for adult and juvenile idiopathic inflammatory myopathies. *Arthritis Care Res* 2010; 62: 465-72.

Saad CG, Borba EF, Aikawa NE, Silva CA, Pereira RM, Calich AL, et al. Immunogenicity and safety of the 2009 non-adjuvanted influenza A/H1N1 vaccine in a large cohort of autoimmune rheumatic diseases. *Ann Rheum Dis* 2011;70:1068-73.

Sampaio-Barros PD, Andrade DCO, Seguro LCP, Pasoto SG, Viana VST, Ribeiro ACM, et al. Pandemic non-adjuvanted influenza A H1N1 vaccine in a cohort of patients with systemic sclerosis. *Rheumatology (Oxford)* 2018 ;57(10):1725.

Seror R, Ravaud P, Bowman SJ, Baron G, Tzioufas A, Theander E, *et al*. EULAR Sjogren's syndrome disease activity index: development of a consensus systemic disease activity index for primary Sjogren's syndrome. *Ann Rheum Dis* 2010; 69: 1103-9.

Shinjo SK, de Moraes JC, Levy-Neto M, Aikawa NE, de Medeiros Ribeiro AC, Schahin Saad CG, et al. Pandemic unadjuvanted influenza A (H1N1) vaccine in dermatomyositis and polymyositis: immunogenicity independent of therapy and no harmful effect in disease. *Vaccine* 2012; 31:202-6.

Schoels M, Aletaha D, Funovits J, Kavanaugh A, Baker D, Smolen JS. Application of the DAREA/DAPSA score for assessment of disease activity in psoriatic arthritis. Ann Rheum Dis 2010;69:1441–7

van den Hoogen F, Khanna D, Fransen J, Johnson SR, Baron M, Tyndall A, et al. 2013 classification criteria for systemic sclerosis: an American College of Rheumatology/European league against rheumatism collaborative initiative. *Arthritis Rheum* (2013) 65(11):2737–47.

Vitali C, Bombardieri S, Jonsson R, *et al*. Classification criteria for Sjögren's syndrome: a revised version of the European criteria proposed by the American-European Consensus Group. *Ann Rheum Dis* 2002; 61: 554-8.

White, N.J., et al. COVID-19 prevention and treatment: a critical analysis of chloroquine and hydroxychloroquine clinical pharmacology. *PLoS Med* 2020;17(9):e1003252-e1003252.

Ye C, Cai S, Shen G, et al. Clinical features of rheumatic patients infected with COVID-19 in Wuhan, China. *Annals of the Rheumatic Diseases* 2020; 79:1007-13.

Zhang Y, Zeng G, Pan H, Li C, Hu Y, Chu K, et al. Safety, tolerability, and immunogenicity of an inactivated SARS-CoV-2 vaccine in healthy adults aged 18-59 years: a randomised, double-blind, placebo-controlled, phase 1/2 clinical trial. *Lancet Infect Dis* 2020:S1473-3099(20)30843-4.

Park JK, Lee MA, Lee EY, Song YW, Choi Y, Winthrop KL, Lee EB. Effect of methotrexate discontinuation on efficacy of seasonal influenza vaccination in patients with rheumatoid arthritis: a randomised clinical trial. *Annals of the rheumatic diseases* 2017;76(9):1559-65.

Miyakis S, Lockshin MD, Atsumi T, Branch DW, Brey RL, Cervera R, et al. International consensus statement on an update of the classification criteria for definite antiphospholipid syndrome (APS). *J Thromb Haemost* 2006; 4(2):295-306.

Rudwaleit M, Landewé R, van der Heijde D, et al. The development of Assessment of SpondyloArthritis international Society classification criteria for axial spondyloarthritis (part I): classification of paper patients by expert opinion including uncertainty appraisal. *Ann Rheum Dis* 2009;68(6):770–6

Rudwaleit M, van der Heijde D, Landewé R, et al. The development of Assessment of SpondyloArthritis international Society classification criteria for axial spondyloarthritis (part II): validation and final selection. *Ann Rheum Dis* 2009;68:777–83.

Aletaha D, Neogi T, Silman AJ, Funovits J, Felson DT, Bingham III CO, Birnbaum NS, Burmester GR, Bykerk VP, Cohen MD, Combe B. 2010 rheumatoid arthritis classification criteria: an American College of Rheumatology/European League Against Rheumatism collaborative initiative. *Arthritis & rheumatism* 2010;62(9):2569-81.

Petri M, Orbai AM, Alarcon GS, Gordon C, Merrill JT, Fortin PR, et al. Derivation and validation of systemic lupus international collaborating clinics classification criteria for systemic lupus erythematosus. *Arthritis Rheum* 2012;64(8):2677–86.

ten Wolde S, Breedveld FC, Hermans J, Vandenbroucke JP, van de Laar MA, Markusse HM, et al. Randomised placebo-controlled study of stopping second-line drugs in rheumatoid arthritis. Lancet 1996;347:347-52.

Tillett W, Costa L, Jadon D, Wallis D, Cavill C, McHUGH JE, Korendowych E, McHUGH NE. The ClASsification for Psoriatic ARthritis (CASPAR) criteria–a retrospective feasibility, sensitivity, and specificity study. *The Journal of rheumatology* 2012;39(1):154-6.

Smolen JS, Aletaha D, Bijlsma JW, Breedveld FC, Boumpas D, Burmester G, Combe B, Cutolo M, De Wit M, Dougados M, Emery P. Treating rheumatoid arthritis to target: recommendations of an international task force. *Annals of the rheumatic diseases* 2010;69(4):631-7.
