# Supplementary material for: Immunogenicity decay and case incidence six months post Sinovac-CoronaVac vaccine in autoimmune rheumatic diseases patients
Source: Nat Commun. 2022 Oct 3;13:5801. doi: 10.1038/s41467-022-33042-0 (PMC9527375; doi:10.1038/s41467-022-33042-0)
Supplement: Supplementary file 3 — Reporting Summary [file 41467_2022_33042_MOESM3_ESM.pdf]

nature research

Corresponding author(s): Eloisa Bonfa

Last updated by author(s): Apr 19, 2022

Reporting Summary

Nature Research wishes to improve the reproducibility of the work that we publish. This form provides structure for consistency and transparency in reporting. For further information on Nature Research policies, see our Editorial Policies and the Editorial Policy Checklist.

Statistics

For all statistical analyses, confirm that the following items are present in the figure legend, table legend, main text, or Methods section.

n/a	Confirmed	
[x]	[ ]	The exact sample size (n) for each experimental group/condition, given as a discrete number and unit of measurement
[ ]	[x]	A statement on whether measurements were taken from distinct samples or whether the same sample was measured repeatedly
[ ]	[x]	The statistical test(s) used AND whether they are one- or two-sided<br>*Only common tests should be described solely by name; describe more complex techniques in the Methods section.*
[ ]	[x]	A description of all covariates tested
[x]	[ ]	A description of any assumptions or corrections, such as tests of normality and adjustment for multiple comparisons
[ ]	[x]	A full description of the statistical parameters including central tendency (e.g. means) or other basic estimates (e.g. regression coefficient) AND variation (e.g. standard deviation) or associated estimates of uncertainty (e.g. confidence intervals)
[ ]	[x]	For null hypothesis testing, the test statistic (e.g. F, t, r) with confidence intervals, effect sizes, degrees of freedom and P value noted<br>*Give P values as exact values whenever suitable.*
[x]	[ ]	For Bayesian analysis, information on the choice of priors and Markov chain Monte Carlo settings
[x]	[ ]	For hierarchical and complex designs, identification of the appropriate level for tests and full reporting of outcomes
[x]	[ ]	Estimates of effect sizes (e.g. Cohen's d, Pearson's r), indicating how they were calculated

Our web collection on statistics for biologists contains articles on many of the points above.

Software and code

Policy information about availability of computer code

Data collection	The analyzed data were extracted from the patients' electronic medical records (PRONTMED) and Study data were collected and managed using REDCap electronic data capture tools (10.5.0 - © 2021 Vanderbilt University) hosted at our Institution.<br>Data collection for ELISA was performed using Indirect ELISA, LIAISON® SARS-CoV-2 S1/S2 IgG, DiaSorin, Italy (Cat# 311450 and Cat# 311451). Neutralizing antibodies were performed using SARS-CoV-2 sVNT Kit, Cat# L00847-A (GenScript, Piscataway, NJ, USA)<br>No custom software codes have been developed.<br>RT-PCR for SARS-CoV-2 analysis. Naso- and oropharyngeal swabs were obtained whenever the participants reported COVID-19 related symptoms, and SARS-CoV-2 RT-PCR was performed using the SuperScriptTM III Platinum® One-Step Quantitative RT-PCR System Cat#11732-088 (Invitrogen, Carlsbad, CA, USA). A m2000sp equipment (Abbott, Des Plaines, IL, USA) was for RNA extraction and a m2000rt (Abbott, Des Plaines, IL, USA) for RNA amplification and detection. The assay was carried out according to the protocol reported by Corman and colleagues as previously reported assay
Data analysis	All statistical analyses were performed using Statistical Package for the Social Sciences, version 20.0 (IBM-SPSS for Windows. 20.0. Chicago, IL, USA). For incident-density data, MedCalc version 20.015 was used.

For manuscripts utilizing custom algorithms or software that are central to the research but not yet described in published literature, software must be made available to editors and reviewers. We strongly encourage code deposition in a community repository (e.g. GitHub). See the Nature Research guidelines for submitting code & software for further information.

Data

Policy information about availability of data

All manuscripts must include a data availability statement. This statement should provide the following information, where applicable:

- Accession codes, unique identifiers, or web links for publicly available datasets
- A list of figures that have associated raw data
- A description of any restrictions on data availability

All the background information on controls and clinical information for the patients with ARD in this study are included in the Source Data provided with this paper (https://figshare.com/xxxxxxx). Raw data for each figure or table have been deposited as a Source Data file. All data used in these analyses can be found at https://figshare.com. (https://figshare.com/s/b25763a08f5d782f50ce). The raw personal data are protected and are not available due to data privacy laws.

Field-specific reporting

Please select the one below that is the best fit for your research. If you are not sure, read the appropriate sections before making your selection.

[x] Life sciences [ ] Behavioural & social sciences [ ] Ecological, evolutionary & environmental sciences

For a reference copy of the document with all sections, see nature.com/documents/nr-reporting-summary-flat.pdf

Life sciences study design

All studies must disclose on these points even when the disclosure is negative.

Sample size	This study is a long-term follow-up from de first study "Immunogenicity and safety of the CoronaVac inactivated vaccine in patients with autoimmune rheumatic diseases: a phase 4 trial" which included sample size calculation. No specific sample size calculation was performed for the endpoints of the present study.
Data exclusions	No data were excluded from the analyses.
Replication	This is an ongoing human trial and therefore there was still no attempt of replication.
Randomization	This was an observational study with no randomized intervention. All participants (patients and controls) received the same vaccine, without experimental groups.
Blinding	This phase 4 prospective controlled observational study with no randomized intervention. All participants (patients and controls) received the same vaccine, without placebo group. Therefore, blinding was not performed.

Reporting for specific materials, systems and methods

We require information from authors about some types of materials, experimental systems and methods used in many studies. Here, indicate whether each material, system or method listed is relevant to your study. If you are not sure if a list item applies to your research, read the appropriate section before selecting a response.

Materials & experimental systems

n/a	Involved in the study	
x		Antibodies
x		Eukaryotic cell lines
x		Palaeontology and archaeology
x		Animals and other organisms
	x	Human research participants
	x	Clinical data
x		Dual use research of concern

Methods

n/a	Involved in the study	
x		ChIP-seq
x		Flow cytometry
x		MRI-based neuroimaging

Human research participants

Policy information about studies involving human research participants

Population characteristics	Male and female individuals with autoimmune rheumatic disease and volunteers (control group) ≥ 18 anos. Participants originated from a large single center (Sao Paulo, Brazil) phase 4 controlled prospective study (no. NCT04754698, CoronavRheum) of immunogenicity and safety of two doses of Sinovac-CoronaVac vaccine in ARD patients and controls (CG) 13. After applying the exclusion criteria, the final study groups consisted of 828 ARD patients and 207 controls vaccinated with 2 doses. ARD group included patients with: 27.5% (n=228) rheumatoid arthritis, 24.9% (n=206) systemic lupus erythematosus, 23.7% (n=196) axial spondyloarthritis, 6.2% (n=51) primary vasculitis, 4.8% (n=40) idiopathic inflammatory myopathies, 4.3% (n=36) systemic sclerosis, and 4.3% (n=36) primary Sjögren's syndrome. Regarding ARD current therapy at 6 weeks after the 2nd dose (D69) the most frequently used were: 62.0% (n=513) immunosuppressive drugs, 36.5% (n=302) prednisone and 35.7% (n=296) biologic therapy. ARD and CG groups were comparable regarding median current age , female sex and Caucasian race.
Recruitment	Autoimmune rheumatic disease (ARD) patients from the Outpatient Rheumatology Clinics at Hospital das Clinicas HCFMUSP, Faculdade de Medicina, Universidade de Sao Paulo, Sao Paulo, SP, Brazil. All participants were > 18 years old, and the controls were balanced with patients by sex and for age (up to 5 years differences) at the entry (1 control: 4 patients). Well-controlled medical conditions were allowed in the CG, except ARD, use of immunosuppressive drugs or HIV infection. All ARD patients received the inactivated Sinovac-CoronaVac vaccine (Sinovac Life Sciences, Beijing, China, batch #20200412) first dose between February 9th and 17th 2021, and the second dose between March 9th and 17th 2021 (28 days apart). No strategy for immunogenicity improvement was applied. The exclusion criteria at vaccination included previous vaccination with any SARS-CoV-2 vaccine, previous history of anaphylactic events after vaccine administration, history of previous vaccination with live virus up to four weeks, or with inactivated virus vaccine up to two weeks, COVID-19 related symptoms, acute febrile illness, Guillain-Barré syndrome, decompensated heart failure, demyelinating disease, history of blood products administration up to six months before the study start. Hospitalized patients or controls were also excluded.
Ethics oversight	The protocol was conducted according to the Declaration of Helsinki and local regulations and approved by the National and Institutional Ethical Committee of Hospital das Clinicas HCFMUSP, Faculdade de Medicina, Universidade de Sao Paulo, Sao Paulo, SP, Brazil (CAAE: 42566621.0.0000.0068). Written informed consent was obtained from each participant before enrollment.

Note that full information on the approval of the study protocol must also be provided in the manuscript.

Clinical data

Policy information about clinical studies

All manuscripts should comply with the ICMJEguidelines for publication of clinical research and a completedCONSORT checklist must be included with all submissions.

Clinical trial registration	ClinicalTrials.gov Identifier: NCT04754698
Study protocol	The protocolo has been submitted.The full trial can be assessed at ClinicalTrials.gov
Data collection	The final study groups for immunogenicity analyses consisted of 828 ARD patients and 207 healthy controls. Blood collections for this study were performed six weeks after thethde 2nd dose (April 19th 2021, D69) and 6 months after the 2nd dose (September 18th 2021, D210) for ARD and CG. All participants signed the written informed consent prior to the vaccination and blood collection. Compensation for participation was not provided. Study protocol availability, data collection and outcomes are listed in the Supplementary Information.<br>A rigorous follow-up of incident cases was performed for all participants with COVID-19 symptoms from vaccine first dose to 6 months after the second dose (D210). Four in-person visits (vaccine first dose, vaccine second dose, D69 and D210) were performed, with a careful checking of a standardized diary regarding COVID-19 history at each visit. All ARD patients and controls were instructed to communicate any manifestation associated with COVID-19 through telephone, smartphone instant messaging or email. A medical team was available for 24-hours to provide a proper follow-up. Suspicious cases of COVID-19, even if mild, were instructed to seek medical care near the residence and to come to our tertiary hospital to undergo a RT-PCR test for SARS-Cov-2. Participants who were unable to come to our center were instructed to go to an independent laboratory near their home. COVID-19 incident cases were followed from the first vaccine dose to 10 days after the second dose (T1) and thereafter, T2, for the following 170 days (from D40 to D210).<br>These participants were tested using real-time reverse transcriptase-polymerase chain reaction (RT-PCR) test for SARS-CoV-2 by naso- and oropharyngeal swabs.
Outcomes	The primary outcome was humoral immunogenicity assessed by the presence of anti-S1/S2 SARS-CoV-2 IgG 6 months after the 2nd vaccine dose (D210) compared to the same parameter at D69 (6 weeks after 2nd vaccine dose) for decay evaluation. Secondary outcomes were: the presence of neutralizing antibodies (NAb), percent inhibition by NAb and, anti-S1/S2 SARS-CoV-2 IgG GMT. Incident cases confirmed by RT-PCR for SARS-CoV-2 infection were also a secondary outcome.
